# Laser-Based Manufacturing of Ceramics: A Review

**DOI:** 10.3390/mi14081564

**Published:** 2023-08-06

**Authors:** Pudhupalayam Muthukutti Gopal, Vijayananth Kavimani, Kapil Gupta, Dragan Marinkovic

**Affiliations:** 1 Mechanical Engineering, Karpagam Academy of Higher Education, Coimbatore 641021, India; gopal.pm@kahedu.edu.in (P.M.G.); kavimani.s@kahedu.edu.in (V.K.); 2 Mechanical and Industrial Engineering Technology, University of Johannesburg, Johannesburg 2028, South Africa; 3 Faculty of Mechanical Engineering and Transport Systems, Technische Universität Berlin, 10623 Berlin, Germany; dragan.marinkovic@tu-berlin.de; 4 Faculty of Mechanical Engineering, University of Nis, 18000 Nis, Serbia

**Keywords:** ceramic, composite, laser, machining, MMC, sintering

## Abstract

Ceramics are widely used in microelectronics, semiconductor manufacturing, medical devices, aerospace, and aviation, cutting tools, precision optics, MEMS and NEMS devices, insulating components, and ceramic molds. But the fabrication and machining of the ceramic-based materials by conventional processes are always difficult due to their higher hardness and mechanical properties. Therefore, advanced manufacturing techniques are being preferred for these advanced materials, and out of that, laser-based processes are widely used. The benefits of laser fabrication and machining of ceramics include high precision, reduced thermal damage, non-contact processing, and the ability to work with complex geometries. Laser technology continues to advance, enabling even more intricate and diverse applications for ceramics in a wide range of industries. This paper explains various laser based ceramic processing techniques, such as selective laser sintering and melting, and laser machining techniques, such as laser drilling, etc. Identifying and optimizing the process parameters that influence the output quality of laser processed parts is the key technique to improving the quality, which is also focused on in this paper. It aims to facilitate the researchers by providing knowledge on laser-based manufacturing of ceramics and their composites to establish the field further.

## 1. Introduction

Ceramics and their composites, including ceramic-reinforced metal matrix composites (MMCs), are preferably used in conditions where other engineering materials cannot be used. On account of the benefits exhibited by these materials, they have been widely used in high-load, high-wear/friction, and extreme temperature conditions and have grown in reputation for numerous commercial purposes in the biomedical (e.g., dental replacement), aerospace (e.g., engine parts), electronic (e.g., insulators), and other modern engineering (e.g., bearings) sectors [1,2]. Conversely, their advanced characteristics, such as high temperature stability and hardness, make ceramics as complex to process with traditional manufacturing techniques, requiring immense expenditure and energy utilization. The methods generally used for ceramic processing, such as hot pressing, slip casting, and sintering, are found to be expensive, and the energy consumption is also higher [3]. In the past few years, LASER (light amplification by stimulated emission of radiation)-based manufacturing techniques such as laser additive manufacturing, laser machining, etc. have attracted global attention for the fabrication and manufacturing of ceramic parts. In laser machining or manufacturing processes, the laser output characteristics play a critical role in determining the efficiency, precision, and quality of the material processing. Table 1 summarizes the main characters involved in laser output for laser machining or manufacturing.

**Figure 1:**
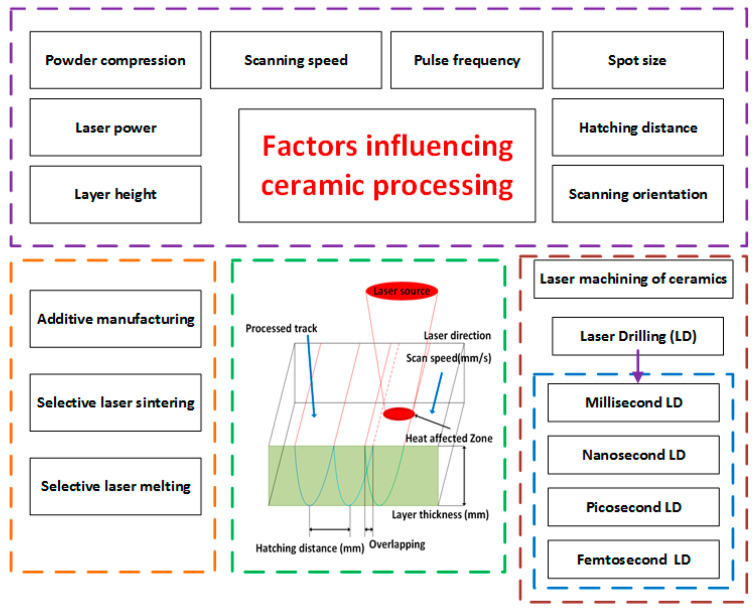
Overview of the laser-based manufacturing of ceramics.

The laser output characteristics provided in Table 1 have to be carefully controlled and optimized based on the specific requirements of the laser machining or manufacturing process. Different applications may require different laser sources and output configurations to achieve the desired results. The ability to tailor and manipulate these laser output parameters allows laser machining to be widely used in industries such as aerospace, automotive, electronics, medical devices, and more. High-power, high-density laser beams in direct additive manufacturing are highly utilized for ceramic processing. Selective Laser Sintering (SLS) and Selective Laser Melting (SLM) are the two major and advanced AM methodologies used for fabricating advanced ceramic parts, which are discussed in detail in this paper. The parameters that influence the output quality of this laser processing technique for ceramics are discussed, along with property improvement practices. Further, laser machining, especially laser drilling of ceramics, is also discussed in detail (Figure 1).

## 2. Laser Fabrication of Ceramics

### 2.1. Selective Laser Sintering

**Figure 2:**
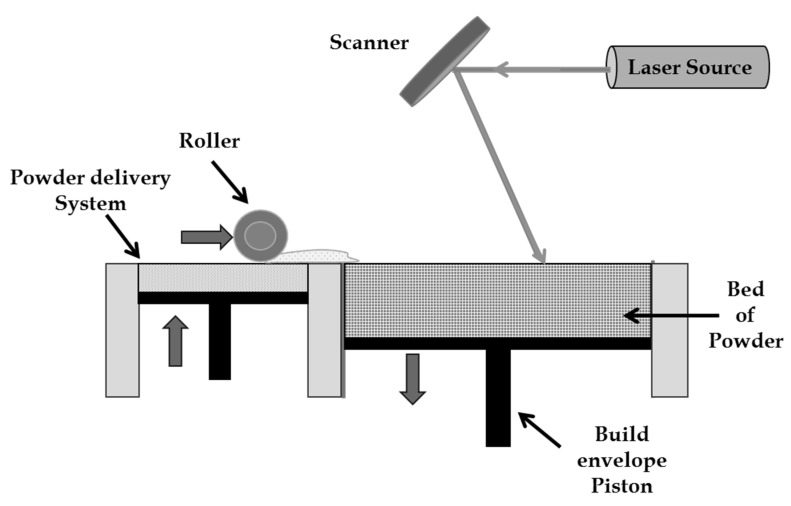
Illustration of selective laser sintering process.

As the name describes, in SLS, metal powders are selectively sintered using a high power laser [4]. The first patent was registered for selective laser sintering in 1986 by Deckard and Beaman of the University of Texas at Austin [5]. Further developments were done by the company named DTM, which was later acquired by 3D Systems in 2001. In SLS, powder is kept in a storage tanker, which is spread over the work table and then sintered with the aid of a laser. Then the bed is moved down, and the next level of powder is spread for a measured height and then selectively sintered depending on the product profile (Figure 2). This process is repeated layer by layer until the complete product or part is manufactured. As the loose powders, i.e., the powders that are not exposed to laser, support the overhanging structures, there is no need for additional supports.

The SLS process is mostly used for preparing models with lower melting points, such as wax, for prototyping at the start. A number of studies were done on SLS using low melting point materials such as polymers initially and later extended to metals, wherein the feasibility of ceramic processing through SLS with alumina particles was reported in 1990 at the University of Texas [6]. Ammonium phosphate with a melting point of 190 °C and boron oxide with a melting point of 460 °C were used as low-temperature binders due to the higher (2045 °C) melting point of alumina. The reason behind the utilization of this feedstock arrangement is that the ceramic, which is also called refractory material, has the tendency to withstand high temperatures. Even though a higher-power laser has the prospect of high temperature generation that is more adequate to trigger the densification process, the time of laser exposure should be high for obtaining pore-free parts, which are extremely hard, and it is very unlikely for ceramic densification. Hence, the methodology of utilizing secondary low melting point binders is followed to fabricate the ceramic parts with acceptable accuracy and density. Therefore, it can be stated that the processing of ceramics through SLS technology is difficult, and hence it is highly necessary to identify the methods to reduce the temperature needed for powder bonding and thus aid densification.

Coating low-melting-point binders over ceramics or mixing them together is a feasible solution for these temperature problems [7]. For bonding, a glassy phase is formed around the ceramic particles when the binder melts during exposure to the LASER. Additionally, these materials are more tolerant of temperature gradients, which is an added benefit. The binder used for this purpose is may be of any material, i.e., either organic [8,9,10] such as polymers or inorganic like metals of low melting points [11,12,13,14,15]. These organic or inorganic binders are removed once the laser processing is completed by way of furnace firing in order to obtain the final ceramic part. In most cases of ceramic SLS processing, the environment during processing is maintained as inert with the aid of inert gases to avoid the oxidation of binding materials. Another problem associated with inorganic binders is that these binders cannot be removed even by furnace firing, and hence they either remain in the final product or form a secondary phase by reacting with matrix powders.

High shrinkage and porosity in the product are the two main problems associated with selective laser sintering of ceramics. It was reported that the density of alumina composite microspheres is less than 50% when they are fabricated by a phase inversion process in which polyamide-12 binder is coated over alumina [16]. Porosity is the only main concern, even though it has good flow characteristics. The reason behind the higher porosity is the space between interspheres that is caused in every step from the spreading of powder in the bed to the final firing in the furnace.

#### 2.1.1. SLS with Infiltration and Pressing

The porous ceramic parts produced through SLS cannot be applied to many applications because better mechanical characteristics can be achieved when the part is fully dense. So, in view of making fully dense ceramic products, either infiltration or isostatic pressing can be applied after SLS [17]. To improve the mechanical strength and density of the parts, some processing can be done at different stages of fabrication. With the additional treatments and processing, ceramic parts of complex shapes with improved densities are successfully fabricated [18,19,20,21,22,23,24]. Shahzad et al. utilized quasi-isostatic pressing (QIP) at elevated temperatures to fabricate alumina parts with Al_2_O_3_–PA composite microspheres as starting materials and achieved 94% theoretical density [16]. Further, the green density is increased from 34% to 83% for Al_2_O_3_–PP composite when both infiltration (with 30 vol% alumina in ethanol) and isostatic pressing (in warm conditions at 135 °C and 64 MPa pressure) are carried out, whereas the final density achieved is improved to 88% from 64%. Similarly, 92% of the theoretical density is achieved through warm isostatic pressing for 3 mol Yttria stabilized zirconia (3YSZ) parts, whereas as much as 35% linear shrinkage is also observed.

By using SiC ceramic filler and PMS pre-ceramic precursors, PDC products are fabricated through selective laser sintering. The density of the prepared parts before and after liquid silicon vacuum infiltration was analyzed, and it was found that the density of the infiltrated parts is almost equal to the theoretical density, while the density of the part before infiltration is half. In addition to this, only minimal linear shrinkage (3%) is observed after firing, while the bending strength increases from 17 MPa for the green part to 220 MPa for the final part. The lower strength of the green part is attributed to high porosity and microcracks in the matrix, while the final parts were almost pore- and crack-free [18].

#### 2.1.2. Laser Processed Ceramics for Biomedical Applications

Selective laser-sintered ceramics have become more and more popular in the biomedical field, such as in the fabrication of intricate and extremely cellular biocompatible personalized scaffolds. These fabrications normally include:

1. High volume fractions of binder of a maximum 60 vol%;
2. Geometrical precision and surface finish are not firmly necessary;
3. Macro-porous configurations are personalized in a convenient way.

Low-melting-point polymers and glasses are utilized in biomedical applications as liquid-phase binders in SLS to assist densification. Hydroxyapatite–tricalcium phosphate, hydroxyapatite–polycarbonate, hydroxyapatite–polyether ether ketone, and silica–polyamide are some examples of bone implants made from a ceramic–polymer mix. Hydroxyapatite–mullite [19,20] and aliphatic-polycarbonate/hydroxyapatite [21] are some examples of biocompatible scaffolds fabricated using ceramic–glass composites.

#### 2.1.3. Factors Affecting the SLS of Ceramics

Ceramics have high melting points, making it challenging to achieve local densification via high-power laser heating in SLS. To facilitate densification, lower melting-point materials can be used as binders by coating or mixing them with the ceramic powder to reduce the required target temperature. Feedstock materials and laser–material interactions are related factors that highly affect the characteristics of the ceramic components produced by SLS. It is noteworthy to mention that the matrix and binder materials should possess good flow characteristics, and the microsized particles of spherical shape possess these properties [22,23]. The green composite’s mechanical performance is also strongly influenced by the binder quantity [24]. In view of the fact that the end product’s porosity is dependent on binder quantity, it is preferred to add a smaller amount of binder. Because of the inconsistent dispersion and segregation of binder in mixed matrix–binder systems, binder-coated matrix powder method parts possess higher strength than mixed matrix–binder systems [25].

In addition to post-processing methodologies like isostatic pressing, feedstock materials with different forms can also be used to improve the density of the laser-sintered part. Tang et al. [26] developed a slurry-like feedstock form instead of conventional ceramic powder form with alumina ceramic and polyvinyl alcohol binder, and by using this system, 98% dense parts are produced. The high distribution and uniformity of the newly developed system are attributed to the higher density. Conversely, it is noteworthy to mention that selective laser sintering is a very intricate process due to the unique interaction between laser beam and material.

The rapid fusing of ceramics in the SLS process affects the microstructures, mechanical properties, and shape of the final components. So the laser energy applied to the ceramic powder bed is the crucial factor in SLS that is directly related to laser power and its scanning speed. The composition of the initial powder and the thermal characteristics of the ceramic material used, like melting point and powder bed packing porosity, are the factors that determine the laser energy requirement. And hence, selecting the proper laser power based on the ceramic material used is very important in SLS.

### 2.2. Selective Laser Melting

The 3D printing methodology that is growing rapidly, predominantly in the area of ceramic forming, is SLM [27]. Simultaneous achievement of properties and part shape and its capability to fabricate tough and robust parts in a single process are attributed to its popularity. The SLM process of ceramics differs from the SLS process in the way that in SLM, the ceramics are melted completely to form solid parts without any additional requirements such as binders. This is because the ceramics are melted fully layer by layer with the aid of a high-density laser that fuses the ceramics together. So, ceramic components of high purity, density, and strength can be manufactured through SLS in minimal time. Further, among the various advanced additive manufacturing processes for ceramics, SLM is the only process that can produce full, dense, ready-to-use ceramic parts of complex net shapes in a single step.

Numerous aspects like properties of initial materials, manufacturing factors, position and orientation of fabrication, interaction during fabrication and its physical and chemical properties, which also include energy source and material interaction, and post-processing affect the overall quality of the part produced through the SLM process. The slice thickness, which is one of the most vital fabrication factors in SLM, influences both productivity and the surface quality of the final part. It is a well-known fact that more slice or layer thickness results in increased roughness and a staircase effect with a lower building time. On the other hand, when less layer thickness is maintained, the surface finish is good, but the build time is high. This most important slice thickness depends on the depth of fusion, which is directly associated with the properties of the material and the interaction between laser and material. Therefore, the optimal parameter combination has to be identified for fabricating a quality part.

#### 2.2.1. Challenges in SLM of Ceramics

There is a small amount of advancement achieved in the ceramic forming field because of the more difficult SLM of ceramics than that of metals and other materials such as composites. Again, it can be stated that the final quality of the SLM part depends on processing parameters. Because there are many chances for problems like the balling effect, which occurs when the energy input is insufficient, the spattering of powder is observed when more energy input is given [28]. On the other hand, direct melting entails a very high temperature reaction between material and power source, i.e., a laser, and a very small interaction time that guides to large temperature gradients within the small amount of material. In SLM, sudden heating and cooling rates while laser scanning result in a very small laser–powder interaction time that induces thermal stresses, which is one of the noteworthy troubles that occur in SLM [29]. These thermal stresses result in the formation of cracks and distortions in sintered components on account of the subordinate thermal shock resistance of the ceramic materials. The same defects are observed when ZrO_2_ ceramic parts are produced through SLM [30]. It is identified from the analysis done to find the source of these thermal stresses that the scanning strategy has a greater effect on thermal stress. And also, it was found that the stress is higher in the direction perpendicular to the laser scanning direction than along it. Another major problem arising due to short interaction times is insufficient melting, which results in huge residual porosity and higher surface roughness in the final component. Only 85% density is reached while fabricating Al_2_O_3_ ceramic parts through SLM. Even though optimized layer deposition and laser scanning parameters were used, deficient material melting results in outsized residual porosity [31]. Similarly, only 56% density, which is very low, is achieved for ZrO_2_–Y_2_O_3_ parts produced through SLM, and further attempts to improve the density, such as heat treatment in a traditional furnace, are also not a success [32].

#### 2.2.2. Modified 3D Printing Methods for Ceramic Processing

Based on selective laser melting, numerous additive manufacturing methods were derived with the capability of processing ceramics, namely slurry-based SLM [33], Laser-Engineered Net Shaping (LENS) [34], and Laser Micro Sintering (LMS) [35]. In order to prevent cracking and lower density of final parts due to lower powder bed density in SLM, other forms of powder packing are tried as an alternative to dry powder deposition. With benefits such as homogeneous and highly packed initial materials in slurry form, there is a reason to be hopeful among the modified techniques. Al_2_O_3_–SiO_2_ parts of 92% density with smooth surface finish are achieved owing to the formation of liquid-phase SiO_2_ when a slurry of Al_2_O_3_–SiO_2_ powder (up to 63 vol%) with water is used as the initial material, which is deposited layer by layer by a doctor blade like in tape casting prior to drying [36]. Fully dense microstructured porcelain parts are also effectively manufactured with later improvements in processes [37].

As the beam moves, the ceramic powder is dropped coaxially to the intended laser spot area so that the immediate formation of a molten pool is achieved in the LENS [38]. Cylinder, cube, and gear-shaped dense Al_2_O_3_ parts with anisotropic mechanical properties were successfully fabricated through LENS by Bella et al. [39]. The density of the developed parts is found to be 94%, and further heat treatments are also not successful in increasing density and altering strength and anisotropy, whereas the grain size increases significantly from 6 to 200 μm. During tensile testing, cracks are formed along the columnar grain boundaries. On the other hand, Niu et al. [40] developed fully dense, plain-shaped, fine-grain-structured Alumina-YSZ components through LENS. As a result of the quick melting/solidification course of action, the 100 nm lamellar colony eutectic spacing is attained. When compared to the traditional directional solidification method of fabricating parts, these parts have comparable mechanical properties, but the surface finish and dimensional accuracy remain inadequate.

Another modified SLM method that can produce ceramic parts with improved resolution and surface finish is LMS [41]. This method differs from the other methods in the way that it uses feedstock materials of submicron size and a Q-switched solid-state, 1 mm-wavelength near-infrared Laser. Fully dense parts of different materials such as alumina and silicon nitride with a few microns of surface roughness and a few tens of microns of resolution are generated through LMS. These surface roughness and resolution values of LMS parts are very low when compared to SLM parts.

### 2.3. SLS vs. SLM

SLS and SLM are both additive manufacturing processes that use lasers to create three-dimensional objects layer by layer. While SLS and SLM have some similarities, they have significant differences in their materials and the underlying mechanisms of working.

Both SLS and SLM are additive manufacturing techniques in which objects are built layer by layer. This allows for the creation of complex geometries that may be challenging or impossible to achieve with traditional manufacturing methods. Both processes utilize a laser as the energy source to selectively fuse or sinter the material powder, solidifying it to create each layer of the object.

In SLS, the material used is typically in powdered form, and the laser selectively heats the powder particles just below their melting point, fusing them together. The final product is a sintered object, where the particles are fused but not fully melted. But in SLM, the laser fully melts the powder particles, resulting in a fully dense and fused object without distinct particle boundaries.

The sintered parts in SLS may have lower density and strength compared to SLM. The presence of unfused particles can result in slightly porous structures. On the other hand, SLM produces fully dense parts, which typically exhibit higher mechanical properties compared to SLS. As a result of the unsintered powder acting as a support structure during the printing process, SLS parts may require post-processing to remove excess powder and, if needed, improve the surface finish. Since SLM produces fully melted and dense parts, there is generally less need for post-processing support removal, and the surface finish is often better compared to SLS.

SLS is commonly used with a variety of materials, including nylon, polystyrene, ceramics, and metals. It is often used for rapid prototyping, functional parts, and low-volume production of end-use parts. SLM is primarily used for metals, including stainless steel, titanium alloys, aluminum, and others. It is widely applied in the aerospace, medical, and automotive industries, where high strength and precision are critical.

## 3. Laser Machining of Ceramics

**Figure 3:**
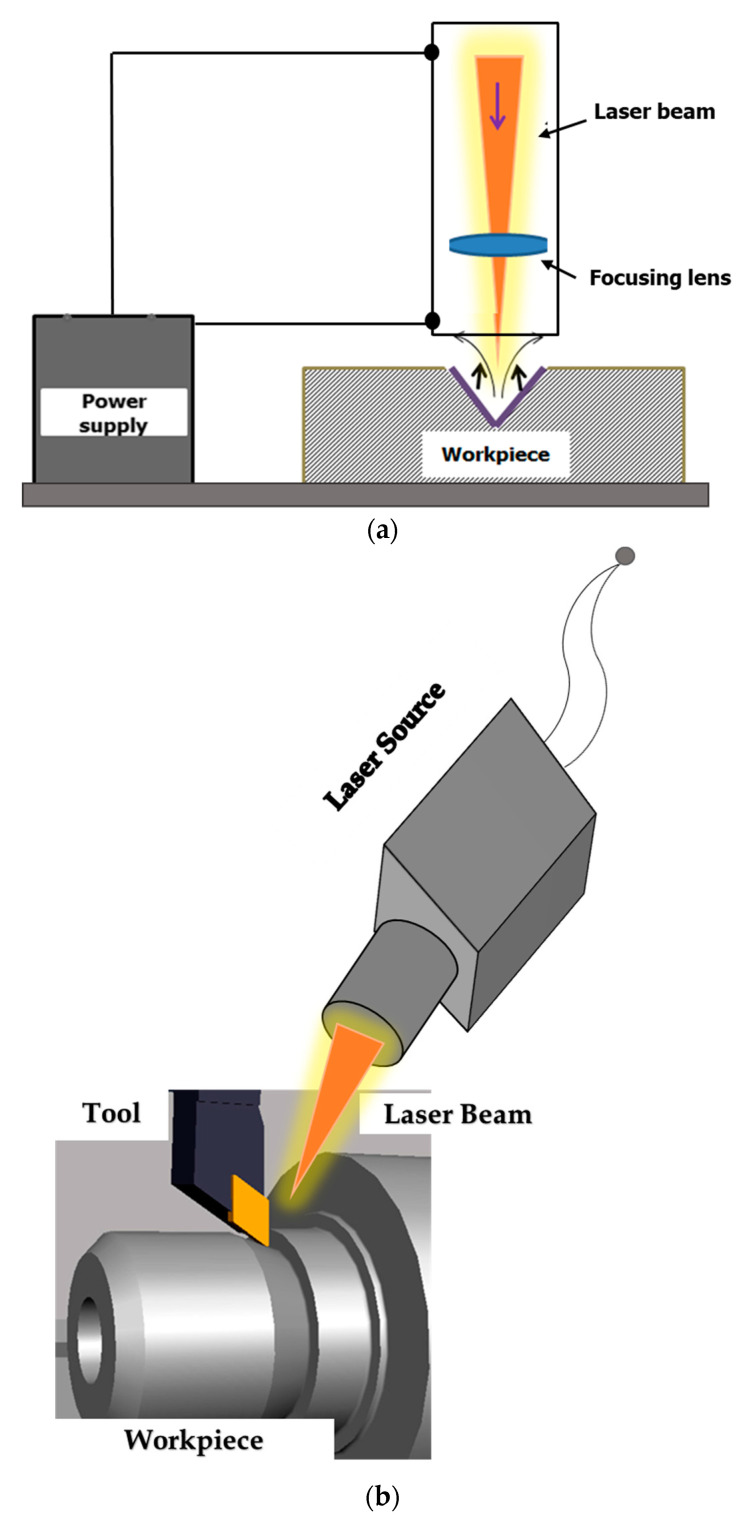
Use of laser in machining, working principles of (**a**) laser beam machining, and (**b**) laser assisted machining.

Any part has to undergo machining at least once before being fed into applications, irrespective of the material type, such as ceramics, metals, etc. Since ceramics are very hard to machine due to their high hardness and brittle nature, Laser Beam Machining (LBM) and Laser Assisted Machining (LAM) are well-suited methods for machining these materials [42,43,44]. In laser beam machining or cutting, a direct laser is used to perform cutting operations such as drilling to make holes and cutting to make intricate shapes and features. Whereas in laser-assisted machining, the assistance of a laser is taken to heat the workpiece material with the aim of facilitating machining (turning and milling) operations [43,44]. The working principles of both LBM and LAM are illustrated in Figure 3 below.

Tsai and Chen [45] proposed a fracture-machining element experimental procedure for laser milling of cavities in ceramic material that can be used for both edge milling and center milling with a reduced amount of power required. It was found by Chang and Kuo while machining alumina ceramics with LAM that there is a reduction in feed force and thrust force of about 22% and 20%, respectively [44]. As a result of the plastic flow of material, the surface obtained through LAM is smooth and straight, whereas the surface produced during conventional machining was rough. An extreme laser beam is used to improve the machinability of the ceramics by disturbing the deformation characteristics of the material [46]. Further, by reducing the mechanical strength, the machinability of ceramics can also be enhanced in LAM [47].

The possibilities of utilizing laser beam machining for a broad variety of materials are investigated through the review by Dubey and Yadava [48], who stated that there is a need for substantial research in LBM of thick materials and microparts. Laser constraints, material characteristics, and process constraints greatly affect the performance of LBM [49]. Experimental analysis on CNT/Fe/Alumina nano-composites machining through laser micromachining revealed that good machining results and no microstructural damage can be obtained when CNT content is high [50].

Even though laser cutting is capable of fabricating cantilever structures in Si_3_N_4_-based composites, their quality is somewhat low, as evidenced by the quality difference in cutting between the laser entrance and exit surfaces [51]. A near infrared ray (NIR) pulsed laser is used to investigate the consequences of different machining conditions in machining Alumina-zirconia composite by Sola and Pena [52]. The temperature of the substrate, pulse number, plasma shield, sample location about the focal plane, and working frequency are the diverse aspects that influence the laser interaction process.

### 3.1. Laser Drilling

Among various laser-based ceramic machining processes, laser drilling is the key process that is extensively used in various applications. Laser drilling yields improved quality with a lesser pulse width and greater peak intensity owing to the changes in the mechanism of material removal. In the laser-based drilling process, thermal penetration depth is equal to diffusion depth, which helps reduce material damage due to lower thermal expansion. Laser drilling processes are classified as laser drilling with milliseconds, nanoseconds, picoseconds, and femtoseconds. Each laser drilling process has specific strengths and is chosen based on the material, hole size, precision requirements, and potential thermal impacts of the application. The selection of the appropriate laser drilling method is crucial to achieving the desired results efficiently and accurately. Millisecond laser drilling is faster for drilling larger holes, while picosecond and femtosecond laser drilling are slower due to their higher precision and intricacy requirements. Femtosecond laser drilling offers the highest precision and minimal heat-affected zones, making it suitable for cutting-edge micro and nano drilling applications. Nanosecond and picosecond drilling offer varying degrees of precision based on application requirements. Millisecond and nanosecond laser drilling may cause considerable heat accumulation and thermal damage. In contrast, picosecond and femtosecond laser drilling minimize heat effects, making them ideal for delicate materials and high-precision applications. Millisecond and nanosecond laser drilling find applications in industries like automotive and aerospace, where speed and efficiency are essential. Picosecond and femtosecond drilling are prevalent in microelectronics, medical devices, and cutting-edge research where precision and minimal damage are critical.

#### 3.1.1. Millisecond Laser Drilling

Laser drilling studies over silicon carbide materials were conducted by Sciti et al. [53] with varying operational parameters and pulse durations of 0.5 to 2 milliseconds (ms). They observed that these hard ceramics can be removed by several reactions that involve vaporization and melting. Microholes in the range of 200 µm were achieved by them over the ceramic surface. Their results reveal that increased pulse timing tends to form cylindrical drill holes, and a higher energy density of the laser tends to form dendrites over the ceramic surface. The influence of the laser drilling parameter over alumina was investigated by Kacar et al. [54], with the pulse duration in the range of 0.5 to 8 ms. They observed that variation in pulse duration and peak power controls taper angles in drilling operations. Likewise, the dimension of outlet holes changes equivalently with respect to pulse timing at constant peak power; however, this mechanism does not occur at the inlet holes. Further, an increment in pulse timing initiates higher re-solidification over the material surface. Laser Drilling of alumina with a pulse duration of 1 to 6 ms was performed by Hanon and his co-workers [55] with varying drilling thickness. Their result reveals that the incremental order of pulse duration increases the hole depth near the inlet of the surface. Further observe the occurrence of columnar grain structures over the surface of holes; however, cooling down in the drilling process initiates crack formation in the ceramic surface. Murray et al. [56] performed drilling operations over partially stabilized tetragonal zirconia and observed that the use of a plasma system reduced the formation of cracks and recasts by decreasing the level of thermal gradients. Further revelations reveal that the thermal properties of ceramic play a key role in ceramic material processing. At a drilling temperature of 1300 °C, the introduction of localized plasma heating resulted in an additional 14% average decrease in microcracking of the recast layer. A spatter-free laser drilling operation was adopted by Guo and a co-worker [57] to perform drilling over alumina-based ceramic material at 0.2 ms of pulse duration. They observed that the incremental increase in pulse number increased the hole depth. Also, it was suggested that adopting this method would deliver homogenous quality finished parts. Low-pressure water jet-assisted laser drilling was carried out by Lu and his research group [58] for drilling alumina ceramic with various drilling parameters. Results reveal that the use of water jet assisted drilling decreases the chance of recast and micro-crack formation over the machined surface of ceramic material. Further, it was observed that gas pressure and pulse energy are the major influencing parameters for laser drilling operations. It was stated that the rate of material removal could reach 90%. Lee and Cheng [59] performed laser circular cutting for drilling operations over alumina and observed that the taper of drilling holes was greater than the attained circular cutting, however, this process consumed less time. It was also found that utilization of auxiliary gas pressure ranging from 20 psi to 50 psi yielded better results.

#### 3.1.2. Nanosecond Laser Drilling

A laser-based drilling operation was performed by nedialkov and his co-worker [60] with pulse duration of 6 ns over aluminum nitride. They investigated the influence of pulsed irradiation on aluminum nitride and observed that a lower laser threshold results in the formation of irregular hole fractures over the drilled surface. The influence of drilling parameters such as frequency, scanning speed, laser power, and hole diameter over alumina was investigated by Bharatish and team [61]. They observed that an increase in laser power increased the hole’s entrance circularity, but an increase in hole diameter showed decremental trend in entrance circularity. Whatever the circularity of the exit, it increases with respect to both hole diameter and laser power. Further, it was observed that heat-affected zones can be controlled by increasing the frequency of the pulse while maintaining a constant laser power source. The effect of drilling parameters over taper and circularity of Titanium nitrate alumina ceramics was examined by Biswas and co-researchers [62]. They observed the effect of drilling parameters such as focal length, air pressure, lamp current, and pulse frequency and attained the optimal parameter for output response. They found that the frequency of the pulse and lamp current act as the governing parameters for output response. In some studies, liquid assisted laser processing has been effectively applied for nanosecond laser operations. For drilling, Silicon carbide liquid-assisted laser processing was adopted by Iwatani and co-workers [63]. Further, it was observed that the incremental order of pulse numbers exhibits an incremental range in hole depth in drilling operations, and it was also observed that increasing the range in water film thickness decreases the etching rate of hard ceramic material. They also observed that underwater laser drilling operations produce holes without the formation of debris particles. Wee and co-workers [64] investigate the influence of cooling medium-based parameters in laser drilling of silicon carbide. They compared the influence of cooling mediums such as alcohol, flow, and stagnant water in a normal environment on the machining quality of laser drilling operations. Results reveal that the usage of cooling medium decreases the redeposition of materials near the processing zone. During the drilling operation, the material removal takes place by melting the material with bubble formation while the cooling medium is superheated. Also depicted is that the use of alcohol-based cooling mediums showcases better performance when compared to other cooling mediums.

#### 3.1.3. Picosecond Laser Drilling

A picosecond-based laser drilling operation was performed over carbon/silicon carbide ceramic by Wang and co-workers [65]. Trepanning and helical drilling operations were carried out by them to produce holes in ceramic material. The result reveals that incremental increases in laser scanning speed decrease the hole depth. Liu and his research group [66] adopted the single and helical ring lines scanning method for performing laser drilling operations over carbon/silicon carbide ceramic. Their results depict that the occurrence of micropores and bubble pits takes place in helical ring mode. Likewise, observation of hole depth and debris reveals higher oxygen content; this might be due to the adopted process parameter. They also observed that increasing energy density increases the hole’s depth and decreases the hole’s exit circularity. Optimization of the effective parameter for picosecond-based laser drilling operations was performed over carbon/silicon carbide ceramic by Zhang et al. [67]. Results reveal that the smaller helical line spacing and width initiate better quality holes in laser drilling operations. However, the machining operational time of the process has been difficult to predict owing to the plasma and debris of the laser process.

#### 3.1.4. Femtosecond Laser Drilling

Lie and a co-worker [68] performed a femtosecond laser drilling operation over alumina. This research group validated the trepanning drilling method and inferred that enhanced quality of holes can be achieved by a femtosecond-based infrared laser when compared to a UV-based nanosecond laser due to its various mechanisms of laser ablation. Femtosecond-based laser drilling operations over titanium carbide were carried out by Zhang and co-workers [69] by adopting helical drilling without cooling conditions viz. air. Results reveal that an increment in laser energy density increases the hole depth and eliminates the parallel grooves over the titanium carbide surface. They found that the repetition rate was lower over the circular ring geometry of the hole. A cooling system based Femtosecond laser drilling operation was performed over silicon carbide by Li et al. [70]. Alcohol was utilized by them as the coolant liquid source, and they observed that the usage of alcohol-based coolant helps in boosting clean and deeper holes over silicon carbide. They observed that the quality of holes attained by a coolant-based system was better when compared to an air-assisted laser drilling operation. Further benefits of coolant-assisted drilling operations were that the rate of processing of holes could be quicker by means of chemical reactions that occur near the drilling surface and the elimination of irradiation. In the present day, chemical-based selective etching operations are effective for higher material removal rates with better flexibility and precision. Hence, the combination of laser with selective chemical etching was considered the better processing method for performing drilling operations with better surface quality and material removal rates.

## 4. Conclusions

A detailed discussion on various important aspects, along with a review of some important past attempts on laser-based fabrication and machining of ceramics and composites, has been done in this paper. Advanced manufacturing processes, mainly SLS, SLM, and laser drilling, are the major focus. The difficulties in SLS of ceramics are the temperature and density of the sintered part. Coating low-melting-point binders over ceramics or mixing them together is suggested as a feasible solution for temperature problems in SLS. In addition to post-processing methodologies like isostatic pressing, feedstock materials with different forms can also be used to improve the density of the laser-sintered part. The overall quality of the part produced through the SLM process depends on numerous aspects like the properties of the initial materials, manufacturing factors, position and orientation of fabrication, interaction during fabrication, and its physical and chemical properties. While laser drilling is performed for ceramics, improved quality is attained when a smaller pulse width and a greater peak intensity are used. Future studies are required to engineer the desirable properties of parts and components using laser-based manufacturing, sustainability aspects of laser fabrication and machining such as life cycle engineering and analysis, and optimization of laser additive manufacturing and machining of ceramics, etc.

## 5. Outlook

The future of laser processing of ceramics lies in its continuous evolution and integration into various cutting-edge industries. As laser technology continues to advance, we can expect further improvements in processing speed, precision, and versatility. With higher-powered lasers, the processing of ceramics will become more efficient, enabling faster production and reducing manufacturing costs. The development of compact and portable laser systems may also pave the way for on-site or in-field ceramic processing, benefiting industries such as construction, archaeology, and cultural preservation. Moreover, the ongoing research in laser-material interactions will expand the range of ceramics that can be effectively processed, including those with unique properties and compositions that were previously challenging to work with. As a result, lasers will find applications in diverse sectors, such as space exploration, renewable energy, and even cutting-edge electronics like flexible and wearable devices. Furthermore, the integration of artificial intelligence and machine learning algorithms into laser processing systems will enhance automation and real-time process optimization. “Intelligent” laser systems will adapt to varying ceramic materials and geometries, minimizing human intervention and maximizing production efficiency. As laser processing becomes more established, specialized training and education programs will likely emerge to cultivate a skilled workforce capable of harnessing the full potential of this technology. This will further drive innovation and the adoption of laser processing techniques across industries. The future of laser processing of ceramics is brimming with possibilities. From revolutionizing manufacturing processes to enabling breakthroughs in advanced technologies, lasers will continue to shape the world of ceramics, leading us into an era of unparalleled precision, efficiency, and ingenuity.

## Figures and Tables

**Table 1:** Laser output characters.

Laser Output Character	Description
Wavelength (λ)	The laser’s wavelength determines the depth of penetration into the material and the efficiency with which the material absorbs the laser energy. For example, certain materials may strongly absorb laser light at a specific wavelength, making them ideal for precise cutting or engraving.
Power (P)	The total energy emitted per unit of time. Higher laser power allows for faster material removal or processing, but it must be controlled carefully to avoid damage to the workpiece.
Beam Profile	Describes the spatial intensity distribution of the laser beam. The shape of the beam profile affects the energy distribution over the processed area, which can influence the accuracy and quality of the machining. Common profiles include Gaussian and top-hat.
Divergence	Refers to the spreading of the laser beam as it propagates through space. Low divergence is desirable for focused, precise material processing.
Pulse Duration	For pulsed lasers, the pulse duration is the duration of each laser pulse. Short pulses can improve material processing precision, while longer pulses might be more suitable for certain applications like Surface Cleaning and Preparation.
Coherence	The property of the laser beam is where all photons oscillate in phase with each other. Coherence is important for certain applications that involve interference effects like holography, laser ablation patterning, and surface structuring.
Spatial Mode	Characterizes the distribution of the laser’s electric field in the transverse plane. Different spatial modes can influence the quality of the focused beam and the precision of material removal.
Polarization	Describes the orientation of the electric field vector of the laser beam. Polarization affects how the laser interacts with certain materials and optical components.
Spectral Width	The range of wavelengths present in the laser emission. A narrow spectral width is preferred for certain precision applications, while a broader spectrum might be suitable for others, like surface cleaning and treatment.
